# Resource‐based trade‐offs and the adaptive significance of seasonal plasticity in butterfly wing melanism

**DOI:** 10.1002/ece3.11309

**Published:** 2024-05-01

**Authors:** Andrew M. Stoehr, Katelyn Glaenzer, Devin VanWanzeele, Samantha Rumschlag

**Affiliations:** ^1^ Department of Biological Sciences Butler University Indianapolis Indiana USA; ^2^ U.S. EPA Great Lakes Toxicology and Ecology Division Duluth Minnesota USA

**Keywords:** butterflies, melanism, phenotypic integration, phenotypic plasticity, seasonal polyphenism, trade‐offs, tyrosine

## Abstract

Phenotypic plasticity is the ability of an organism to alter its phenotype in response to environmental cues. This can be adaptive if the cues are reliable predictors of impending conditions and the alterations enhance the organism's ability to capitalize on those conditions. However, since traits do not exist in isolation but as part of larger interdependent systems of traits (phenotypic integration), trade‐offs between correlated plastic traits can make phenotypic plasticity non‐ or maladaptive. We examine this problem in the seasonally plastic wing melanism of a pierid (Order Lepidoptera, Family Pieridae) butterfly, *Pieris rapae* L. Several wing pattern traits are more melanized in colder than in warmer seasons, resulting in effective thermoregulation through solar absorption. However, other wing pattern traits, the spots, are less melanized during colder seasons than in warmer seasons. Although spot plasticity may be adaptive, reduced melanism of these spots could also be explained by resource‐based trade‐offs. Theory predicts that traits involved in resource‐based trade‐offs will be positively correlated when variation among individuals in resource acquisition is greater than variation among individuals in resource allocation strategies, and negatively correlated when variation in allocation is greater than variation in acquisition. Using data from both field studies and laboratory studies that manipulate dietary tyrosine, a melanin precursor, we show that when allocation to thermoregulatory melanism (ventral hindwing, and basal dorsal fore‐ and hindwing “shading”) varies substantially this trait is negatively correlated with spot melanism. However, when there is less variation in allocation to thermoregulatory melanism we find these traits to be positively correlated; these findings are consistent with the resource‐based trade‐off hypothesis, which may provide a non‐ or maladaptive hypothesis to explain spot plasticity. We also show that increased dietary tyrosine results in increased spot melanism under some conditions, supporting the more general idea that melanism may involve resource‐based costs.

## Graphical abstract

Predictable seasonal plasticity may be adaptive. However, traits that are developmentally correlated with other plastic traits may change in predictable but non‐adaptive ways. We show patterns of butterfly wing melanism plasticity that are consistent with resource‐based trade offs and may not be adaptive.

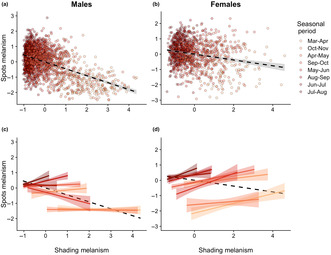

## 1 INTRODUCTION

One of the ways organisms cope with environmental variation is through adaptive phenotypic plasticity, i.e. through adaptive behavioral and/or developmental plasticity in response to cues experienced at one time (and place) that serve as reliable predictors of future environmental conditions (Pfennig, 2021a; Via et al., 1995). Such plasticity is adaptive if the net fitness of plastic individuals, across the variable environment, is higher than that of non‐plastic individuals (Doughty & Reznick, 2004). Strictly speaking, however, phenotypic plasticity is not an attribute of individuals but instead of traits because traits vary in whether they are plastic, how plastic they are, and to what environmental factors they respond. In addition, not every instance of plasticity can be assumed to be adaptive (Doughty & Reznick, 2004; Ghalambor et al., 2007; Pfennig, 2021b). In response to environmental variation, therefore, most organisms will show plasticity in multiple traits, some of which might be cases of adaptive plasticity but some of which might be neutral or even maladaptive. These latter cases of non‐adaptive plasticity may be by‐products of trade‐offs due to phenotypic integration, i.e. the fact that multiple traits may rely to varying degrees on underlying common cue perception and developmental mechanisms and may compete for the same resources (Pigliucci, 2004; Schlichting, 1989). The purpose of this article is to explore one such case of plasticity in multiple traits where the adaptive significance of some of this plasticity is unclear. We use a field study and a laboratory experiment to consider adaptive plasticity and trade‐offs from ecological and mechanistic perspectives, a valuable integrative approach to understanding organismal biology (Garland et al., 2022).

**FIGURE 1:**
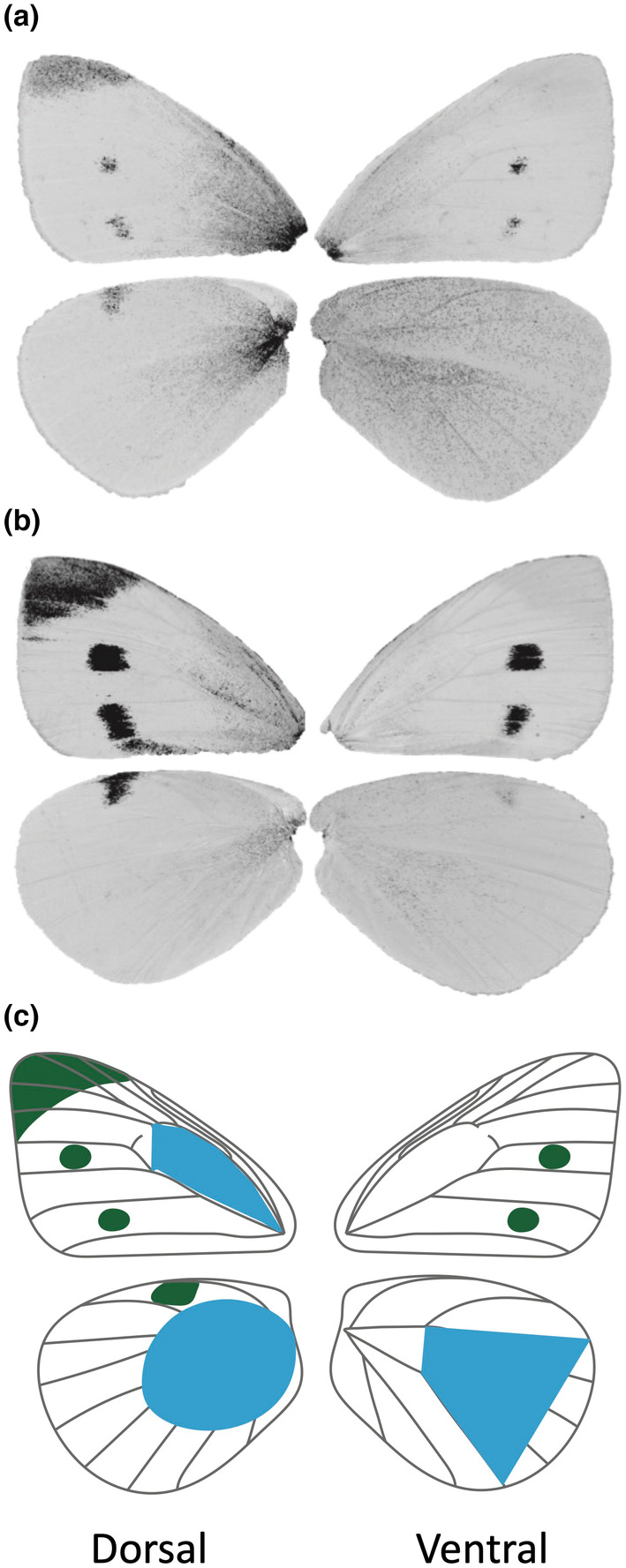
Wing pattern variation in the cabbage white butterfly (*Pieris rapae* L.). In cool‐season butterflies (a top) the dorsal forewing tip patch and dorsal and ventral wing spots (denoted in green in c, bottom) are small, whereas the basal portions of the dorsal fore‐ and hindwing and ventral hindwing surfaces are more heavily melanized with a diffuse “shading” of black scales (denoted in blue in c, bottom). In warm‐season butterflies (b, middle) wingtip patches and spots are large but the dorsal basal and ventral hindwing regions are less melanised. All melanised traits were quantified as the area falling above a threshold of 150, on a 0–255 (white to black) grayscale. Wingtip patches, spot (“spots” in this article) sizes, and dorsal hindwing melanism were quantified in their entirety, whereas basal dorsal forewing and ventral hindwing melanism were quantified as the area falling above the threshold in the polygon defined by wing‐vein landmarks as shown. The total area of the ventral hindwing polygon served as a proxy for overall wing size. See text for details.

Particularly well‐studied cases of phenotypic plasticity, especially in insects, include those cases where seasonal cues (temperature and/or photoperiod) experienced early in development affect the phenotypes expressed quite some time later, i.e. seasonal polyphenisms (Nylin, 2013; Shapiro, 1976; van der Burg & Reed, 2021). Among the most compelling cases of adaptive phenotypic plasticity are the seasonal melanisms in some temperate pierid butterflies (Order Lepidoptera, Family Pieridae) (Kingsolver, 1995a, 1995b; Shapiro, 1976; Watt, 1969). In these species, butterflies that emerge from the chrysalis (i.e. eclose) in the spring and autumn, when temperatures are relatively low, have wing pattern traits on the ventral hindwing and basal portions of the dorsal fore‐ and hindwings that are more heavily melanized (i.e. relatively more black scales) than those of butterflies eclosing during the warmer summer months (Figure 1a). Through solar absorption, these darker individuals can more effectively thermoregulate under the lower ambient temperatures they experience, whereas the less‐melanized summer phenotypes are less prone to overheating during higher summer temperatures (Ellers & Boggs, 2004; Kingsolver & Watt, 1983; Watt, 1968, 1969). Melanism of these particular wing pattern traits — hereafter “shading” — in pierid butterflies is therefore a form of adaptive phenotypic plasticity and offers some of the best support for the “thermal melanism hypothesis” (Clusella Trullas et al., 2007).

In these same butterflies other melanin‐based wing pattern traits, particularly the black spots, wing‐tip patches, and distal marginal bands, also vary seasonally suggesting that this too may be a form of adaptive plasticity (Hoffmann, 1973; Hovanitz, 1945; Kingsolver & Wiernasz, 1991; Shapiro, 1976; Stoehr & Goux, 2008). However, these traits are generally *less* melanized in cool‐season butterflies compared to warm‐season butterflies, which have more heavily melanized margins, spots, and wing‐tip patches (Figure 1b). Because the spots, wing‐tip patches, and margins — hereafter the “spots group traits” or simply “spots” — vary seasonally in a pattern *opposite* to that of the melanized shading traits, the plasticity of the spots is harder to understand as a form of adaptive plasticity, at least for thermoregulation based on solar absorption. One hypothesis that could explain the opposing patterns of seasonal variation of these different melanin‐based wing‐color traits is that of resource‐based trade‐offs. If there are resource costs to producing heavily melanized traits then increased melanization of some traits (i.e. the thermoregulatory‐important shading traits) in the spring and autumn may come at the expense of decreased melanization of other traits (i.e. the spots group traits). In the summer, when less melanization of the shading is necessary, greater melanization of the spots would be possible. Such resource‐based trade‐offs might be particularly acute in butterflies because as herbivorous animals they may be nutrient‐limited (Elser et al., 2000; Slansky & Feeny, 1977) and as holometabolous animals, they largely acquire resources in a separate (e.g. larval) stage from that during which those resources are allocated (e.g. the pupal stage) (Morehouse, 2014). We are implicitly assuming that all else being equal, larger spots/patches are favored. Because there is evidence that these traits may play a role in sexual signaling, this is not an unreasonable working assumption (Stoehr et al., 2016; Wiernasz, 1989); however, we address the possibility that seasonally varying sexual selection, or other forms of selection (e.g. predation) could vary seasonally as well in Section 4.

**FIGURE 2:**
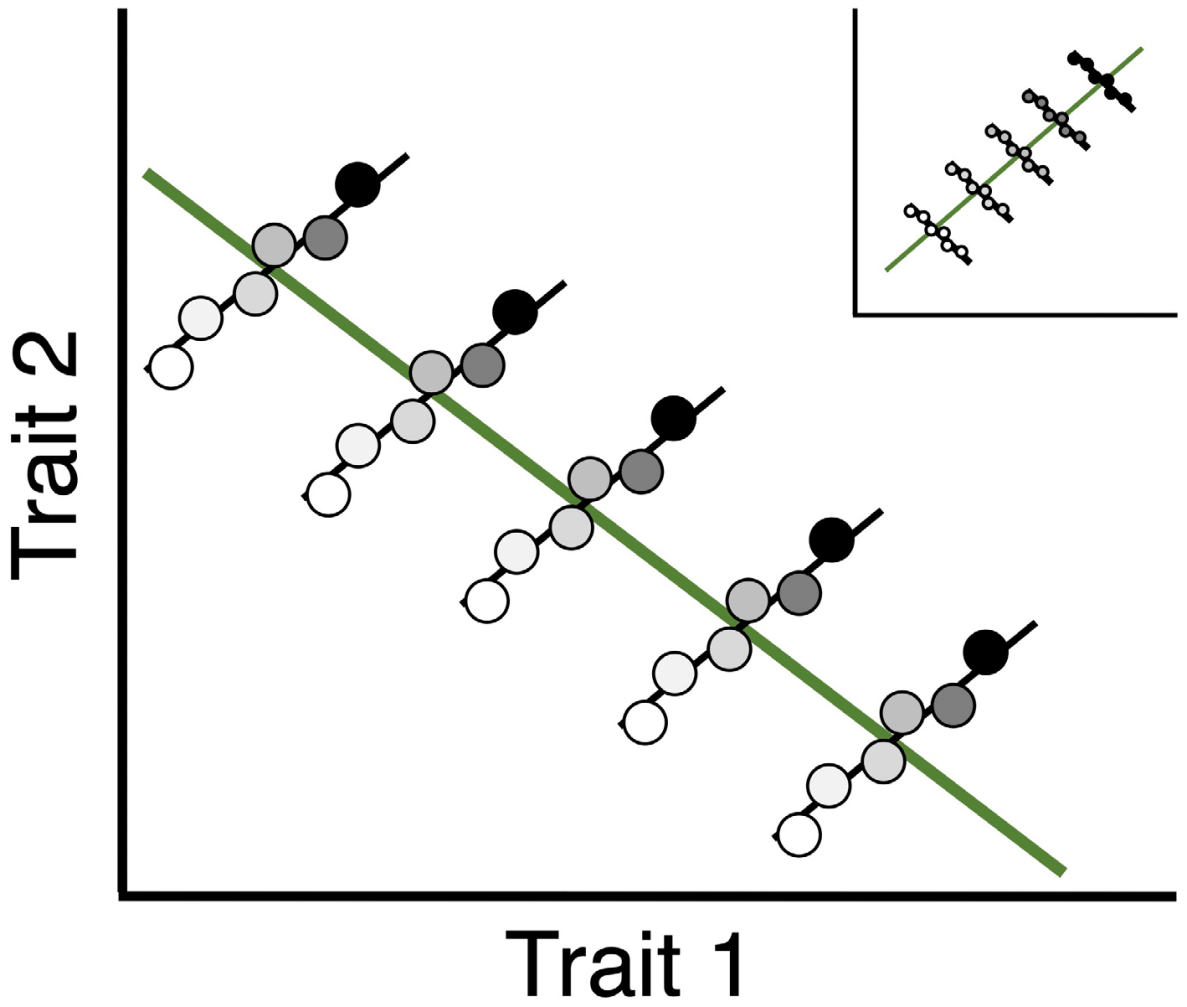
Resource‐based trade‐offs may produce within‐group correlations between competing traits that differ in sign from across‐group correlations. If groups differ considerably in resource acquisition the across groups correlation may be positive despite trade‐off‐induced negative correlations within groups (inset). However, if differences among groups in allocation to traits are large the across‐groups correlation may be negative despite positive within‐group correlations due to variation in resource acquisition (main body). In this figure, darker points represent individuals that have acquired more resources.

In this article, we examine this resource‐based trade‐off hypothesis through studies using both wild‐caught butterflies and manipulations of resources in laboratory‐reared butterflies. In part, we rely on the theory developed to understand why trade‐offs in life‐history traits are often not found when they are expected, but we apply these ideas to the wing‐pattern traits instead. The life‐history theory posits that because organisms may be resource limited we would predict trade‐offs, manifested as negative correlations, between traits that compete for these common resources (e.g., reproduction versus maintenance, offspring size vs. number, etc.) (Roff, 1992; Stearns, 1992). Despite the simple logic of this prediction, not only are such negative correlations sometimes not found but traits hypothesized to be competing for resources are sometimes uncorrelated or even positively correlated. The “Y‐model” of resource allocation, made most widely known by van Noordwijk and de Jong (1986), provides a simple but elegant solution to this observation: some individuals acquire more resources than others and those individuals can therefore invest relatively more in all those traits despite that these traits are still competing for resources. More specifically, the fundamental insight of van Noordwijk and de Jong (1986) is that whether we observe negative or positive (or no) correlations between the traits may depend on whether individuals vary more in the amount of resources they acquire or if they vary more in how they allocate those resources to competing traits (Metcalf, 2016). Most often, this model is used to show how positive correlations between traits across aggregated individuals result from what may be “invisible” negative correlations between traits within disaggregated groups of individuals varying considerably in resource acquisition (Figure 2, inset). However, the converse also follows: if individuals across the aggregate vary considerably in how much they allocate resources to competing traits the negative correlation can result even if it is the result of more positive trait correlations at some disaggregated level because within these groups some individuals still acquire more resources than others (Figure 2, main body). More generally, this pattern whereby the direction of the relationships between variables may change depending upon the level (i.e. aggregated or disaggregated) of analysis is sometimes referred to as Simpson's (or sometimes Robinson's) paradox (Kievit et al., 2013).

In the cabbage white butterfly (*Pieris rapae* L.) melanism varies primarily in response to the temperature (and perhaps to a lesser degree, the photoperiod) experienced by the developing larvae and pupae (Stoehr & Wojan, 2016). The flight season is long: in our central Indiana (USA) populations butterflies appear as early as mid‐March and individuals of the last adult generation are observed as late as early November, and there are several generations per year (Shull, 1987). As a result of this long flight season and the seasonal, temperate climate of Indiana, the developing butterflies experience considerable temperature variation and therefore allocation to melanism varies considerably as well when we consider all the individuals occurring across this long flight season. At the same time, however, over shorter seasonal periods allocation to melanism is expected to be relatively less variable because the temperature varies less on much shorter timescales. In other words, the temperatures experienced by developing butterflies (and therefore allocation to melanism) vary much more among butterflies from March to November than do temperatures experienced by butterflies within any given month, assuming that seasonal temperature variation is reasonably correlated with the actual temperatures experienced by the developing butterflies. We can use the trade‐off hypothesis to make the following predictions: Across the entire flight season (mid‐March to early November) variation among individuals in allocation strategies should be relatively large, with some individuals (cooler months) allocating more resources to thermoregulatory shading melanism traits and others (warmer months) less so. As a result, when analyzed at this (aggregated, i.e. across all butterflies) level spots melanism and shading melanism should be negatively correlated due to their reliance on common resources. However, within a given shorter period (e.g. 30 days) when temperatures vary less, allocation to melanism should likewise vary less and as a result, variation among individuals in resource acquisition should take on greater importance in determining trait correlations; therefore the correlations should be less negative or even positive. In short, the trade‐off hypothesis predicts that the negative correlation across the season can be understood to be a result of less negative/more positive within‐group correlations (Figure 2). Note, however, that the predictions do not require that the “groups” be real groups with hard boundaries, only that the relative variation among individuals in resource allocation versus resource acquisition differs. Indeed, we would expect the correlations between traits to shift from negative to positive as the period (and hence experienced temperature range) analyzed changes from long (e.g. aggregated across the entire flight season) to progressively shorter.

We also addressed similar predictions in lab‐reared butterflies by experimentally manipulating rearing conditions (temperature and photoperiod) to induce variation in allocation to melanism and simultaneously manipulating dietary tyrosine content to induce variation in resource acquisition. The lab experiment therefore serves as another test of the resource‐based trade‐off hypothesis but allows us to more directly control the variables of interest. We manipulated tyrosine because tyrosine is the amino acid precursor from which melanin is ultimately synthesized (Sugumaran, 2002; True, 2003; Whitten & Coates, 2017). Although tyrosine can be synthesized from phenylalanine, this synthesis itself may be costly (Fuchs et al., 2014). We discuss the potential costs of melanin synthesis in more detail in the Section 4, below, but a recent and thorough review of the evidence for dietary effects on and costs of animal melanism can be found in Britton and Davidowitz (2022).

For our laboratory experiments, we can make the following predictions derived from the same trade‐off hypothesis: Within a given dietary treatment, but across rearing conditions (i.e. cool‐short vs. warm‐long temperature/photoperiod treatments) spots and shading melanism should be negatively correlated. This prediction follows because among individuals melanism allocation should vary considerably (because of the temperature/photoperiod rearing conditions), whereas resource acquisition should vary less since all individuals received the same amount of resources. This prediction is analogous to the prediction from our analysis of all butterflies across the entire flight season in the field study. In contrast, within a given temperature/photoperiod rearing treatment but across diets the traits should be more positively correlated. This prediction follows because the tyrosine manipulation should increase relative resource acquisition variation among individuals but melanism allocation variation should be relatively less given identical temperature/photoperiod conditions. This prediction is analogous to the prediction from our analysis within shorter temporal periods in the field study. Last, we might also predict that increased tyrosine would allow the butterflies to produce larger spots, with the effect more pronounced in those reared under lower temperature/shorter photoperiod conditions when spots are smaller.

## 2 MATERIALS AND METHODS

### 2.1 Wild‐caught butterflies

Butterflies for the first study were captured in nets from a variety of locations in Hamilton and Marion counties, two adjacent counties in central Indiana, and within distances of approximately 9 km or less from a position at 39.91° N, −86.15° W. We sampled butterflies opportunistically, i.e. we did not use a standardized sampling protocol, except that attempts were made to capture butterflies regularly throughout the flight seasons (i.e. March through November) in 2015–2021. We captured a total of 3395 butterflies during this period, with a mean of 485 ± 208 (standard deviation) butterflies per year, with some years including as few as 168 (2018) and others as many as 776 (2016). Of these 3395 butterflies, 63.2% (2147) were males and 36.8% (1248) were females. The male bias was consistent (though variable, from 58 to 77%) in all periods except the latter half of October/first half of November when males made up 47.7% of captured butterflies. As soon as butterflies were captured they were either placed into empty plastic containers and soon frozen or they were placed immediately in kill jars with ethyl acetate. Butterflies were stored in a freezer. Later, each wing was removed at the point of attachment with small scissors and all four wings were stored in glassine envelopes until we photographed them.

We photographed the dorsal and ventral wings of each butterfly using a Canon EOS Digital Rebel T3i with a Canon EF 100 mm macro lens and Canon MR‐14EX macro ring flash, all mounted on a copy stand perpendicular to the wings such that the flash surface was 31.65 cm above the wing. The flash was set to ¼ power, shutter speed to 1/200 s, f‐stop to F16, and ISO to 100. From the digital images, we measured the following six “spots group traits” from each butterfly: the black wingtip patch on the dorsal forewing, the anterior and posterior spots of the dorsal and ventral wing surfaces, and the spot on the anterior edge of the dorsal hindwing (Figure 1c, green). We also measured three thermoregulatory‐relevant “shading” traits: the basal melanization of the dorsal forewing discal cell as defined by wing veins and wing‐vein intersections as depicted, the basal melanization of the dorsal hindwing in a region of interest that captured all of the melanism in that area, and the melanization of a pre‐defined ventral hindwing polygon (Figure 1c, blue). The total area of that ventral hindwing polygon also served as a proxy for overall wing size (and is hereafter referred to simply as “wing size”), which we use as a covariate in our statistical models because the melanized traits may covary with wing size. All of the traits were quantified in pixels and then converted to mm^2^. To quantify the area of melanization we converted the images to 8‐bit grayscale, with 0 as white and 255 as black, and used a preselected threshold of 150 to separate black wing scales from white or yellow scales. We selected this threshold after viewing several images across the entire grayscale to identify the threshold that best appeared to separate black scales from the others; this threshold was then used for all specimens. Previous work has shown that similar methods result in highly repeatable measurements and also that the ventral hindwing polygon is a good proxy for overall wing size (Stoehr & Wojan, 2016). Image analysis was conducted in ImageJ (Schneider et al., 2012) with the customizable plugin ObjectJ (Vischer & Nastase, 2009).

We then created two new composite variables, hereafter “shading melanism” or simply “shading” and “spots melanism” or simply “spots”, from the measured wing pattern elements. Shading melanism was the sum of the values for three traits shown in blue in Figure 1c. We chose these three traits because experimental work has shown that melanism in these wing regions affects body temperature via solar absorption (Wasserthal, 1975; Watt, 1968, 1969). Spots melanism was the sum of all of the other traits, i.e. the wingtip patch, the dorsal and ventral forewing spots, and the hindwing spot (Figure 1c, green). These traits were chosen because they are seasonally plastic (being reduced in size in the cooler months) and, except the ventral spots, have been hypothesized to function in thermoregulation through an alternative mechanism to solar absorption called “reflectance basking” (Kingsolver, 1985a), which we discuss further in the Section 4.

To confirm that shorter periods experience less temperature variation, we first considered the range of temperatures across the flight season of 15 March to 15 November (because very few butterflies are captured or observed outside of these dates), a period of approximately 246 days, depending upon the year. We then considered several shorter periods. In our analyses and figures, we focus primarily on eight approximately month‐long periods (i.e. ~30 days) starting from 16 March to 15 April, and so on up through 16 October to 15 November. However, to confirm that our assumptions about temperature variation (and our conclusions from the wing melanism analyses) do not depend upon the precise choice of temporal periods, we consider several other periods. The shortest periods consisted of approximately 15‐day periods, defined as days one through 15 and day 16 to the last day of each month. In addition to the approximately one‐month‐long periods described above, we also considered just each month (e.g. June, July, etc). Still longer, we considered periods of approximately 45 days (defined 16 April to 31 May, 1 June to July 15, etc.), two approximately 60‐day periods (one which combined the latter half of a given month with the following month plus the first half of the next month, e.g. 16 March through 15 May, etc. and another that simply combined months, e.g. April and May, June and July, etc.), and two approximately 90‐day periods (one of March through May, June through August, September through November and the other of April through June and July through September). These different time periods are reported as “approximate” durations because our choices are sometimes necessarily truncated periods. For example, we did not consider dates prior to 16 March or after 15 November. We used temperature data from the National Centers for Environmental Information (NCEI) of the National Oceanic and Atmospheric Administration (NOAA) Global Summary of the Day (GSOD) files for the weather station at the Indianapolis International Airport (72438093819). While it is important to acknowledge that microhabitat‐level variation in temperature experienced by the developing butterflies was unknown, and possibly important (Woods et al., 2015), we emphasize that in this study we are trying to address consistent and clear seasonal‐level variation in melanism; we assume, therefore, that our considerations of temperature variation have been done at a reasonable scale.

### 2.2 Laboratory experiment (tyrosine supplementation & temperature/daylength manipulations)

In this experiment, we crossed a dietary tyrosine supplementation treatment (“low” vs. “high” tyrosine) with a temperature/photoperiod (warm, long‐day vs. cool, short‐day) rearing treatment, starting with 1200 individual larvae (caterpillars) haphazardly assigned evenly across diet treatments (600 each) and biased slightly in favor of the cool, short‐day treatment (720 to cool, short‐day, 480 to warm, long‐day). The butterflies used in this experiment were taken from a larger lab colony that had been maintained continuously for approximately four or five generations, the founders of which were originally purchased from Carolina Biological Supply (Burlington, North Carolina, USA). Butterflies were induced to lay eggs on Parafilm (Bemis, Neenah, WI, USA) which we then cut into strips containing approximately 20–40 eggs each (Webb & Shelton, 1988). One strip each was placed onto an artificial diet in 190 individual plastic (59.15 mL or 2 fluid ounces) cups and maintained at 25°C, 16 L:8D. To maximize survival we allowed the larvae to feed on the diet we use for standard colony maintenance (“Maintenance Diet”; see Table S1) until they were approximately 1 cm long, which usually means the early 3rd larval instar; the vast majority (>95%) of a larva's resources is consumed in the final two, i.e. 4th and 5th, instars (Theunissen et al., 1985). At this point we haphazardly assigned larvae to pairs, matched approximately (i.e. quickly, by eye) by size, and then randomly (by coin flip) assigned each larva in the pair to either a low tyrosine (LT) or high tyrosine (HT) diet. Thus, we had equal numbers of larvae (600) assigned to each diet treatment.

We then haphazardly assigned the 1200 larvae to one of two temperature/day length combinations (25°C, 16 L:8D “warm/long‐day”, hereafter W/L conditions, or 19°C, 8 L:16D “cool/short‐day”, hereafter C/S conditions). Previous work has demonstrated that these rearing conditions result in considerable variation in melanism (Stoehr & Wojan, 2016). We biased this assignment so that 40% (480) were assigned to the warm/long‐day conditions and 60% (720) were assigned to the cool/short‐day conditions because we knew from previous maintenance rearing in the lab that some portion of the cool/short‐day specimens would enter diapause (and some proportion of those would not survive) and some would not. However, we did not know precisely what those proportions would be; the 40:60 bias was chosen somewhat arbitrarily. Ultimately, 633 (88%) of the 720 assigned to the cool/short‐day treatment survived, and of those 45.8% developed directly and 54.2% diapaused. In this study, we limit our presentation to non‐diapausing butterflies because diapausing butterflies require a prolonged chilling period during the pupal stage for successful development and eclosion, which would add an additional treatment to just that subset of cool/short‐day specimens. That said, our main conclusions do not change when we consider those butterflies as well.

Both diets had half of the casein replaced with cellulose (non‐nutritive bulk), lowering the protein content of these diets relative to the “Maintenance Diet”. We added 3.0 g of L‐tyrosine to the HT diet. Wheat germ and casein provide most (~75%) of the protein in these diets with the balance provided by other ingredients such as cabbage powder and Torula yeast. The casein and wheat germ each provide approximately 40% of the dietary tyrosine, Torula yeast about 18%, and cabbage powder approximately 2%. We estimate that the low tyrosine (LT) diet provides 1.372 g (or 0.737% of dry ingredients) of tyrosine per batch. The 3.0 g of added tyrosine in the high tyrosine (HT) diet is 2.31% of the mass of the dry ingredients, thus slightly more than triple that of the control low tyrosine diet. Note that both diets, however, provide considerably more tyrosine (as a percent of dry weight) than cabbage (*Brassica oleracea*), a common host plant for *Pieris rapae*. We estimate that 0.24% of the dry mass of cabbage is tyrosine. Cabbage is approximately 16.37% protein, our LT diet is approximately 20% protein and our HT diet is approximately 21.3% protein. As we state these are estimates because we did not analyze the protein or tyrosine contents of the diet. Our goal was not to produce diets of a particular tyrosine content but rather to create two diets that differed substantially in tyrosine content but with similar overall protein contents. Our estimates suggest we achieved this goal. (See Table S1, and Appendix S1 for details about these estimates.)

We maintained the larvae and pupae on the experimental diets and under the temperature/day‐length combinations until adult eclosion. After eclosion, they were frozen, each wing was removed at the point of attachment, and later photographed. Photography and measurement methods for these specimens were identical to those described above for the wild‐caught butterflies. In some instances, wing patterns could not be reliably measured because of wing damage or failure to close. The final sample sizes for the field study of wild‐caught butterflies consisted of 2049 males and 1174 females. In the lab study final sample sizes were as follows: warm/long‐day high‐tyrosine (males: *n* = 105, females: *n* = 40), warm/long‐day low‐tyrosine (males: *n* = 115, females: *n* = 89), cool/short‐day high‐tyrosine (males: *n* = 48, females: *n* = 87), cool/short‐day low‐tyrosine (males: *n* = 66, females: *n* = 85).

#### 2.2.1 Ethics & fieldwork statement

Although large numbers of wild‐caught butterflies were euthanized in this study it is unlikely that our sampling would have negative impacts on the long‐term stability of the population; in addition, *Pieris rapae* is an introduced agricultural pest and thus not a species of conservation concern in our study region. Because butterflies are insects, Institutional Animal Care and Use Committee (IACUC) approval was not required to conduct the research. Nevertheless, reasonable attempts were made to limit the suffering of the butterflies during capture and rapid euthanasia, which was conducted by either freezing or with the use of ethyl acetate in jars.

### 2.3 Statistical analyses

All statistical analyses were conducted in R, version 4.1.2 inside the RStudio 2022.02.0+443 “Prairie Trillium” IDE (for macOS). To confirm that the range of temperatures does indeed depend upon the period length being considered, we calculated the range (maximum minus minimum) for each period (pooled across all years) and then calculated the mean range for periods of varying lengths. We did not conduct formal statistical analyses of these values. To examine the relationships among wing‐pattern traits we used similar approaches for both studies, i.e. the study of wild‐caught butterflies and the experimental laboratory study. We fit linear models with spot size as our response variable, and shading melanism as our predictor while including our wing size proxy (ventral hindwing polygon area) as a covariate to account for the potential relationship between wing size and the extent of melanism. Depending upon the hypothesis being tested the models also included as fixed categorical predictors the shorter periods (in the wild‐caught butterfly study) or the dietary and temperature/photoperiod manipulations as well as some interactions as detailed below. Prior to analysis, we standardized the melanism traits and wing size to mean 0, and standard deviation 1, for two reasons. First, because results can now be interpreted in phenotypic standard deviations our comparisons across experiments and sexes in this study are more general and this should also allow more meaningful comparisons to other studies that examine similar questions in wing pattern melanism but use slightly different methods of measurement. Second, this standardization has some other desirable statistical effects, most notably that we can more meaningfully interpret both main effects and interactions in models with categorical and continuous predictors (Schielzeth, 2010). The homogeneity of variance and normality of residuals for the models was assessed by visually inspecting residuals vs. fitted, scale‐location, and normal Q–Q plots. Because of considerable imbalance in sample sizes F‐tests were conducted using adjusted (i.e. Type 3) sums‐of‐squares with the Anova function from the car package (Fox & Weisberg, 2019) and when the categorical variables were included we used sum contrasts. The values of 95% confidence intervals for simple slope estimates were obtained from the emtrends function and estimated marginal means from the emmeans function, both from the emmeans (formerly lsmeans) package (Lenth, 2016). Degrees of freedom in our statistical analyses reflect the number of butterflies included in each analysis which differs slightly from the total captured or reared because some butterflies were not included when wing pattern traits were too damaged (torn, missing, or stained) to be properly measured.

**FIGURE 3:**
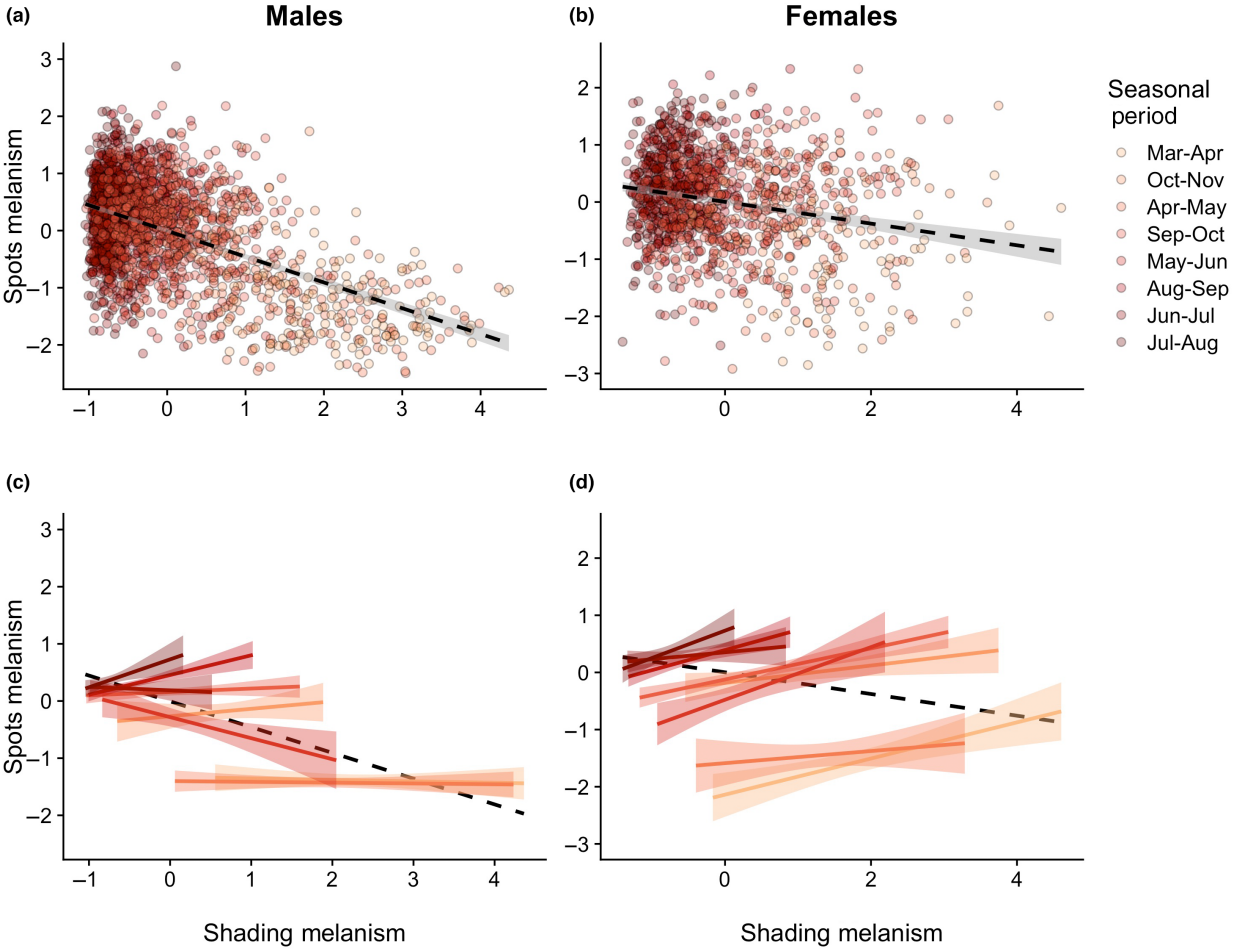
Spots melanism (spot size) as a function of “shading” (i.e. thermoregulation) melanism and seasonal periods. Across the entire flight season, spots melanism declines as shading melanism increases for both males (a) and females (b). However, within a given shorter (~30 days) seasonal period the relationship between spots and shading melanism tends not to decline and in several cases may be positive; this is true for both males (c) and females (d). In (a) and (b), the points are partial residuals controlling for wing size, with values standardized to mean 0, standard deviation 1.0 prior to analysis (see text for details); gray bands around the regression lines indicate 95% confidence intervals. In (c) and (d), points have been eliminated for clarity but within‐period regression lines are shown, along with the 95% confidence intervals. Points (a, b) and lines with bands (c, d) are colored according to the seasonal period to which they correspond, with darker colors being the warmer periods.

Because our models contained a wing size covariate we plotted data that took that into account so the data points in Figures 3 and 5 show the partial residuals from our linear models, holding wing size at the (standardized) mean value of 0 (see Moya‐Laraño & Corcobado, 2008 for a discussion of partial residuals). Partial residuals were obtained from the visreg package using the visreg function (Breheny & Burchett, 2017). Additional R packages used in data analysis and figure preparation include tidyverse packages (Wickham et al., 2019), patchwork (Pedersen, 2022) and cowplot (Wilke, 2022).

### 2.4 Wild‐caught specimens

We tested our prediction that spots melanism, our response variable, would be a decreasing function of shading melanism when considered across all butterflies (i.e. disaggregated) in the flight season by fitting a linear model that included both shading melanism and wing size as continuous predictors. To test the prediction that within‐period slopes would be more positive (i.e. than the slope among all butterflies disaggregated), we fitted a similar linear model that included wing size as a covariate as well as shading melanism but also included the time‐period variable and the period‐by‐shading interaction. Shorter periods were treated as fixed effects because they are being considered in essence as “temperature treatments”. Because of the sexual dimorphism in wing melanism we analyzed females and males separately. We did not consider capture location because all butterflies were captured in ecologically very similar locations within a few kilometers of each other and because we have no detailed information about the developmental history (e.g. specific hostplant, specific microclimate, etc) of the specimens. We pooled across years because our interest is in broad seasonal patterns of plasticity.

Note that our main interest is not in whether these within‐period slopes differ from 0 but rather how they compare to the negative slope when we analyze across the entire flight season. For this reason, our primary means of inference is the comparison of the within‐period slope estimate(s) and confidence intervals (CIs) with that of the analysis across all periods, and we emphasize those particular comparisons of interest for our predictions in the Section 3 below. (Tables showing all *F*‐test results, regression coefficients, and simple slopes are included in the Supporting Information.)

### 2.5 Laboratory experiment (tyrosine & rearing manipulations)

Our trade‐off hypothesis predicts that within a given diet treatment the spots melanism (response variable) should be a negative function of shading because variation in allocation should be relatively large compared to variation in acquisition. To test this we included the diet treatment, shading, the wing size covariate, and the diet‐by‐shading interaction as predictors in a linear model, with spot size as our response variable. Increased tyrosine might alleviate the trade‐off between these traits, resulting in a more negative slope for the low‐tyrosine diet; including the interaction allowed us to consider this possibility. To test the prediction that across diets but within rearing conditions the spots and shading should be positively related we included in a model the rearing (i.e. temperature/photoperiod) treatment, shading, the wing size covariate, and the rearing conditions‐by‐shading interaction as predictors. For reference purposes, i.e. as a comparison to the wild‐caught study, we also show (as black dashed lines in Figure 5) the slope of the fit across all butterflies without considering treatment groups. Lastly, we also examined the estimated marginal means of spot size from this model to assess whether tyrosine supplementation increased spot size. As with the wild‐caught butterflies study, the sexes were analyzed separately, and full statistical results are reported in the Supporting Information tables.

## 3 RESULTS

### 3.1 Wild‐caught butterflies

As one would expect in a temperate seasonal climate such as central Indiana, across the entire flight season temperatures vary widely. Across the entire flight season, the mean daily temperature range was approximately 30°. In other words, days of the colder months were about 30° colder, on average, than days of the warmer months. Each progressively shorter time period we considered experienced a narrower range of temperatures. For example, the shortest period we considered, 15 days, tends to vary by approximately 16.2° on average, whereas the approximately 30‐day periods used in the analyses presented in Figure 3 vary by 19.2°, on average. Each progressively longer period, from 15 to 30 to 45, 60, 90, and 246 days shows a wider average temperature range (Figure S1). That said, warmer periods also tended to be less variable.

**FIGURE 4:**
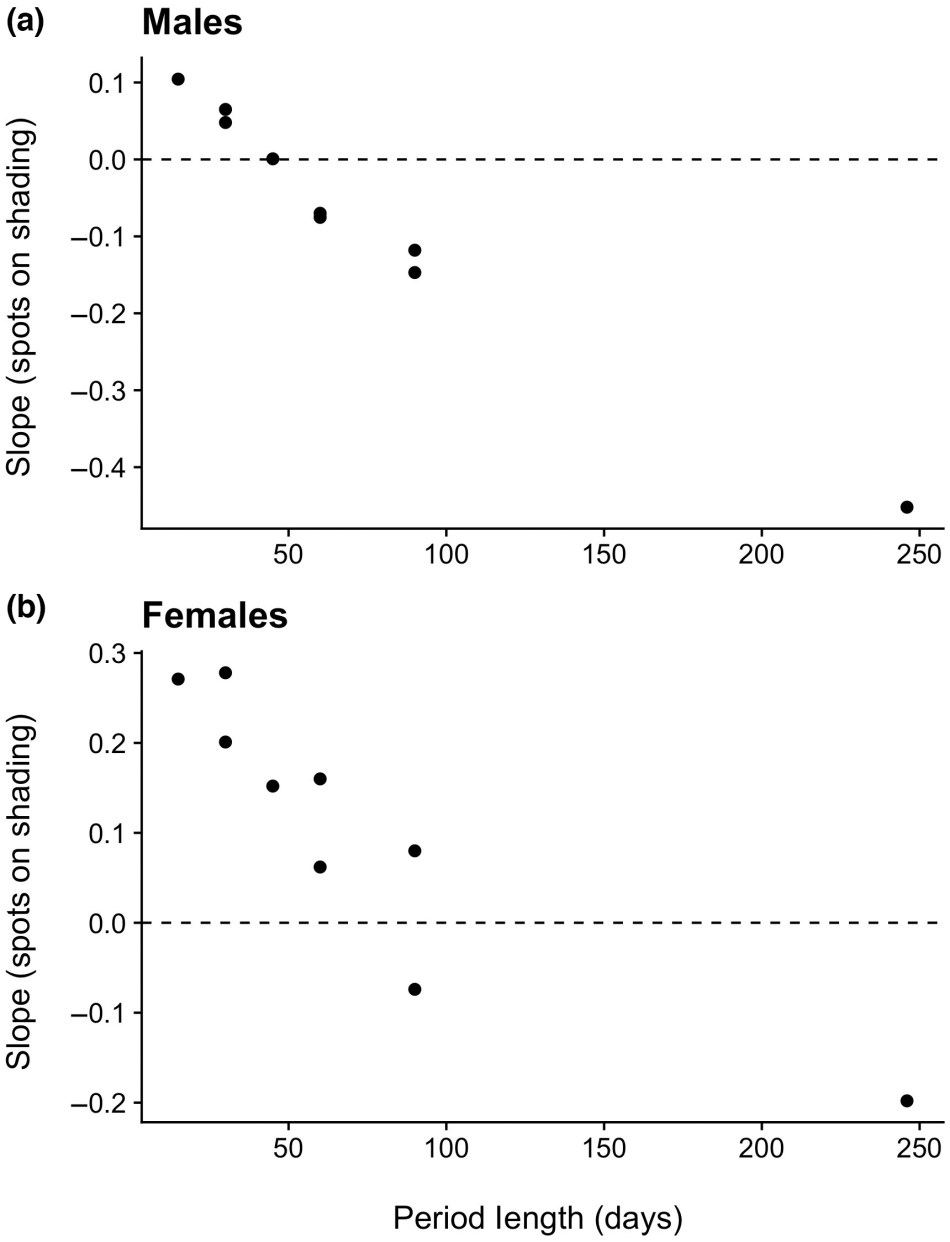
The sign of the relationship between spot size and shading melanism depends upon the length of the seasonal time period under consideration. Slopes (dependent variable) are the grand slopes from linear models modeling spot size as a function of shading that include as predictors seasonal periods from 15 up to 246 days. At short time periods, spots tend to be a positive function of shading melanism (points above the horizontal dashed lines), whereas at longer periods spots tend to be a negative function of shading melanism.

We find that in both male and female butterflies spots melanism is a decreasing function of shading melanism when we consider butterflies captured across the entire flight season from late March to early November, but these negative relationships are the result of a series of more positive within‐period relationships, as predicted by our trade‐off hypothesis (Figure 3). We focus our presentation on the analyses with the approximately 30‐day long periods comprising the latter half of each month plus the first half of the subsequent month (e.g. 16 March to 15 April, etc.) but see also below, and Figure 4. The relationship between spots and thermoregulatory shading melanism across all male butterflies is negative (−0.452, 95% CI: −0.485, −0.419, *F*_1,2046_ = 724.35, *p* < .0001, Figure 3a). However, the grand slope estimate for the shorter period was 0.065 (95% CI: −0.012, 0.142), and the simple slope estimates for four periods (July–August through October–November) were all positive and with 95% CIs that did not overlap the CI of the estimate from across the entire flight season. Slopes for the March–April through June–July periods were negative but only one produced a 95% CI that overlapped the CI of the estimate from across the entire flight season. (See Tables S2a–c and S3a–c for full statistical results.) Variation in within‐monthly period slopes was reflected in an interaction between period and shading melanism (*F*_7,2032_ = 5.166, *p* < .0001).

In wild‐caught females, the relationship between spots and shading melanism across all butterflies was likewise negative (−0.198, SE = 0.0251, 95% CI: −0.248, −0.149, *F*_1,1171_ = 62.58, *p* < .0001, Figure 3b). As in males, the within‐period analysis shows that this negative relationship across all butterflies is also a series of positive within‐period relationships: the grand slope estimate for the period effect was 0.278 (95% CI: 0.197, 0.359), and all of the individual period slope estimates were positive; in only one case (April–May) did the 95% CI overlap with the estimate or CI of the across‐group slope (Figure 3d; Tables S4a–c and S5a–c). In females, the interactive effect of shading melanism and seasonal period on spots melanism was not statistically significant (*F*_7,1157_ = 1.65, *p* = .12).

As our hypothesis predicts, as the time period under consideration became progressively shorter, relative the entire flight season, the relationship between spots and shading melanism becomes progressively more positive (Figure 4). In other words, the relationship between spots and shading melanism is negative when analyzed at the longest time periods, positive when analyzed at the shortest time periods, and tends to approach zero at intermediate time periods. Although the relationship between the slopes and time period of consideration is qualitatively similar for both sexes, it is consistently more positive in females.

### 3.2 Laboratory experiment (tyrosine & rearing manipulations)

**FIGURE 5:**
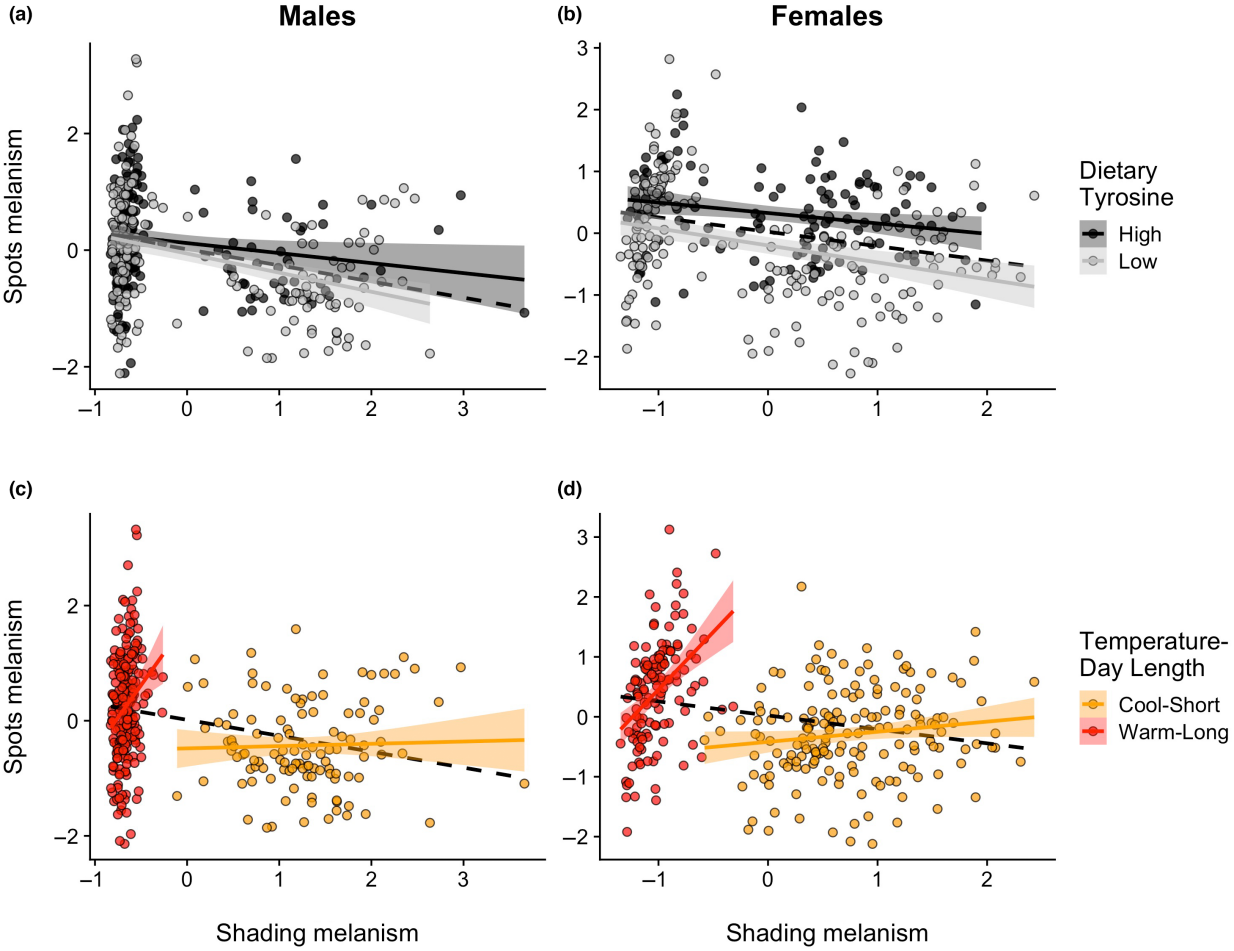
Spots melanism as a function of “shading” (i.e. thermoregulation) melanism, dietary tyrosine content (HT = high tyrosine, LT = low tyrosine), and rearing temperature‐daylength. In (a) and (b), the relationship is shown within each dietary tyrosine treatment but across the rearing‐temperature‐daylength treatments. In (c) and (d), the relationship is shown within each rearing‐temperature‐daylength treatment, but across the dietary tyrosine treatments. Bands depict the 95% confidence intervals. The black dashed line is the regression line across all butterflies without consideration for experimental treatments. Points are partial residuals controlling for wing size and were also standardized to mean 0, standard deviation 1.0, prior to analysis (See text for details).

The results of the laboratory experiments were qualitatively similar to those of the field (i.e. wild‐caught butterflies) study, and qualitatively similar between sexes. In short, we find a negative relationship between spots and shading melanism when considered within each dietary tyrosine treatment, as predicted (Figure 5a,b). However, and as predicted, the melanin‐based traits are generally more positively related when analyzed across diets but within the rearing conditions (i.e. temperature/photoperiod) treatment groups (Figure 5c,d). (Tables S6–S11 report full statistical details.)

More specifically, our hypothesis predicts that within a diet treatment but across rearing conditions, the melanism traits should be negatively related and indeed this is what we find. In males, for both the high‐tyrosine (slope = −0.172, 95% CI: −0.327, −0.016) and low‐tyrosine groups (−0.327, 95% CI: −0.448, −0.206) we found negative relationships, with the slope steeper in the low‐tyrosine group, although the interaction was not statistically significant (*p* = .12) (Figure 5a). The hypothesis predicts that within rearing treatments, but across diets, the melanism traits should be positively related. This was true for the warm‐temperature/long‐day treatment group slope (slope = 2.243, 95% CI: 1.073, 3.413), which was different from zero as well as from the slope from the analysis across all butterflies (i.e. compared to −0.277, 95% CI: −0.372, −0.181, the dashed black line in Figure 5c). For the cool‐temperature/short‐day treatment group, we did not find strong evidence of a positive relationship (slope = 0.040, 95% CI: −0.205, 0.284) though it, too, was more positive than the slope when considered across all butterflies (Figure 5c). The difference in slopes between the two rearing treatment groups is reflected in the interaction (*p* < .001). The estimated marginal mean spot size for high‐tyrosine males was larger than that of low‐tyrosine males, though this difference was not statistically significant (*p* = .07).

The results for females in the tyrosine‐manipulation experiments were qualitatively very similar to what we found for males. Within each diet, but across temperature/photoperiod rearing conditions, we found negative relationships between the melanism traits (high tyrosine: −0.168, 95% CI: −0.332, −0.005; LT −0.275, 95% CI: −0.388, −0.162), as the trade‐off hypothesis predicts. As in males, the slope was steeper for the low‐tyrosine group but again, this interaction was not statistically significant (*p* = .29). Within rearing treatments but across diets, we found a strong positive relationship between traits in the warm‐temperature/long‐day butterflies (slope = 1.920, 95% CI: 1.235, 2.604) and a weaker positive relationship in the cool‐temperature/short‐day butterflies (0.169, CI: −0.023, 0.361). Note that while the 95% CI in the cool‐temperature/short‐day butterflies does contain zero, it does not overlap that of the analysis across all butterflies (i.e. the dashed black line in Figure 5d: slope = −0.233, 95% CI ‐0.330, −0.136). The slopes for the two treatment groups differ (interaction *p* < .0001). Comparison of the estimated marginal means for spot sizes in females shows evidence for larger spots in the high‐tyrosine females (*p* < .0001).

## 4 DISCUSSION

Using both naturally occurring variation in wild‐caught butterflies and direct manipulation of resource abundance and rearing conditions in laboratory experiments we have adopted the framework of the “Y‐model” of resource acquisition versus resource allocation, usually applied to life‐history trade‐offs (van Noordwijk & de Jong, 1986), to explore complex patterns of phenotypic plasticity in butterfly seasonal melanism. We find that when we consider levels of analysis that maximize variation among individuals in how they allocate melanin to “shading” melanism (i.e. the melanism of wing regions that function in solar absorption) those that allocate the most to shading melanism (i.e. colder environmental conditions) have smaller melanized wing spots while those allocating less to shading (i.e. warmer conditions) have larger spots, findings consistent with a resource‐based trade‐off hypothesis. Furthermore, at levels of analysis that minimize relative resource allocation variation compared to resource acquisition variation (i.e. within shorter seasonal periods in wild‐caught butterflies or across dietary tyrosine levels in lab experiments), the negative relationships are reduced in magnitude, eliminated, or even reversed. In fact, this shift in the sign of the relationship between traits from negative to positive as we consider analyses that prioritize variation in resource acquisition over variation in resource allocation among individuals is what the acquisition‐allocation model would predict. In short, we can consider the negative relationships we find between spots and shading melanism to be functions of a series of more positive within‐group relationships, suggesting that the butterflies are in fact “trying” to produce large spots but are constrained in their ability to do so when forced to allocate more melanin to the adaptively plastic thermoregulatory, i.e. “shading”, traits. The increase in spot size under higher dietary tyrosine lends further support to the hypothesis. The general consistency between our findings in both wild‐caught butterflies and laboratory‐reared experimental manipulations is encouraging as well, given the limits of each approach, i.e. unknown developmental histories in the former, and a likely lack of genetic variation in the latter.

Seasonal melanism in *Pieris rapae* spots certainly has many of the hallmarks of potentially adaptive plasticity (Doughty & Reznick, 2004): it consistently varies in response to environmental variation (temperatures experienced during development) that also predicts future environmental variation (temperatures experienced during adulthood). Indeed, Kingsolver's (Kingsolver, 1985a, 1985b, 1987) “reflectance basking hypothesis” provides an adaptive plasticity hypothesis for the seasonal variation of spots. This hypothesis proposes that some pierids, particularly the whitest/most reflective species (e.g. genus *Pieris*), may reflect sunlight from the more distal portions of the wings onto the body when the butterflies bask in the appropriate posture (see also Shanks et al., 2015). Reduced melanism of wing‐tip patches and spots would therefore be advantageous in cool‐season butterflies because this reduced melanism effectively increases the (remaining) reflective surface area of the wings. Kingsolver and Wiernasz (1987) argue that the negative correlations between the shading and spots melanism traits are in fact particularly strong evidence for the adaptiveness of the reduced cool‐season spot sizes given that a reasonable null hypothesis for melanin‐based trait covariation might predict positive correlations among traits based simply on their presumably common underlying developmental mechanisms. Alternatively, or perhaps in addition to a thermoregulatory role, seasonal spot size variation in *Pieris* could reflect seasonal changes in intra‐specific communication (e.g. mate choice) and/or seasonal changes in selection through predation as may occur in some other butterfly species, such as *Bicyclus anynana* (Prudic et al., 2011, 2015; van Bergen & Beldade, 2019).

While we acknowledge the plausibility of these different adaptive hypotheses for spot plasticity in *Pieris*, none has been tested convincingly. Proving that spot variation is not adaptive is probably impossible; a new untested adaptive hypothesis can always be invoked (Gould & Lewontin, 1979). However, because organismal phenotypes are the products of integrated traits that may share underlying developmental mechanisms we should be cautious when attributing adaptive explanations to even the most predictable forms of phenotypic plasticity. Consideration of plasticity in the context of mechanisms and phenotypic integration can help us understand why plasticity in some traits might be understood as the result of trade‐offs (Garland et al., 2022). We see the value of the work we present here as support for, though not irrefutable proof of, a null hypothesis that is arguably more parsimonious than the other suggested adaptive hypotheses for the “reversed” seasonal melanism of the spots in *Pieris* butterflies, and also as a demonstration of why and how we might consider non‐adaptive hypotheses for predictable plasticity. Even more generally, considering trade‐offs at different scales may offer insights into evolutionary and ecological mechanisms that would be less evident from narrower perspectives (Agrawal, 2020).

While we offer the trade‐off hypothesis as a nonadaptive alternative to the reflectance basking hypothesis to explain the plasticity of the wing tip patch and spots these two hypotheses are not necessarily mutually exclusive. For example, the negative correlations among these trait groups might arise because of trade‐offs but then become adaptive through the functional coadaptation mechanisms (Kingsolver, 1987) of the reflectance basking hypothesis as the highly reflective wing surfaces and appropriate basking postures evolve (Kingsolver, 1988). *Colias* species and their relatives (subfamily Coliadinae, sister to the clade containing subfamilies Pseudopontiinae and Pierinae, the latter of which contains the genus *Pieris*; Kawahara et al., 2023; Wahlberg et al., 2014) also may show the decreased dorsal forewing melanization in spring alongside the increased ventral hindwing melanization (and vice versa) (Hoffmann, 1973; Hovanitz, 1945; Shapiro, 1976). However, they typically do not exhibit the highly reflective white “background” coloration (Schmitz, 1994) nor do they typically bask in the “reflectance basking” posture (Kingsolver, 1988). The negative correlations among the “thermoregulatory/shading group” and “spots group” wing pattern elements may be ancestral, which is consistent with the evolutionary scenario proposed above. What might therefore be described as a “constraint”, i.e. the phenotypic integration of the melanin‐based wing pattern traits due to a common currency, may be important in guiding adaptive evolution (Merila & Bjorklund, 2004; Schwenk & Wagner, 2004). Comparative phylogenetic analyses among the pierid butterflies, where changes in background color (i.e. white, yellow), melanism plasticity, host‐plant resource abundance, and possibly other selective factors are all considered simultaneously, could be potentially useful in testing the admittedly speculative evolutionary hypothesis presented above.

Possible costs, or lack thereof, of melanin synthesis and coloration, have been of considerable interest for some time, primarily as they relate to sexual ornaments (Britton & Davidowitz, 2022; Griffith et al., 2006; Gudowska et al., 2022; Guindre‐Parker & Love, 2014; McGraw, 2008; Roulin, 2016; Stoehr, 2006). The question is also of interest among entomologists studying ecological immunology because melanin synthesis is important to the insect immune response (Debecker et al., 2015; Ehrlich & Zuk, 2019; Krams et al., 2016; Moore & Martin, 2016). Evidence for resource costs of melanin synthesis comes from studies that manipulate dietary nutrient content (e.g. host‐plant quality or protein content of artificial diets) and find effects on melanism (see recent review by Britton & Davidowitz, 2022). Although tyrosine can be synthesized from phenylalanine, this synthesis itself may not be cost‐free and there is evidence from both mammals and insects that supplementary tyrosine can affect melanization (Evison et al., 2017; Morris et al., 2002; Watson et al., 2018). Indirect evidence for melanin‐synthesis costs in insects also comes from studies that find negative phenotypic (or sometimes genetic) correlations between various melanin‐based traits such as color and immune defense or between melanin‐based traits and other fitness components (Busso et al., 2017; Kangassalo et al., 2016; Moore et al., 2018; Roff & Fairbairn, 2013; Sun et al., 2018, but see also Sandre et al., 2018). In the present study, increased dietary tyrosine increased melanization of the spots in cabbage white butterflies therefore adding additional support to the hypothesis of costly melanin‐synthesis. However, earlier work in the species examining trade‐offs among different melanin‐based traits, namely immune defense, pupal color, and wing melanization, found only very small effects of dietary nutrients on wing melanism; in addition, these effects were limited to females and showed increased, not decreased, melanism under resource restriction (Stoehr, 2010). However, in that study, only total wing melanism was considered so effects on spots, in particular, were not examined. Melanization of the wings of monarch butterflies also seems resistant to variation in diet quality (Atterholt & Solensky, 2010; Johnson et al., 2014), though not necessarily to other environmental stressors such as parasitism (Alaidrous et al., 2022). Britton and Davidowitz (2022) argue that the literature showing that dietary resource content affects some melanin‐based traits but not others may reflect how melanin synthesis and distribution is prioritized among traits and across species, and suggest further research would be valuable.

The fact that we found that the relationship between spots and shading melanism varied among “groups” is also notable. Generally, we found that the strongest most positive relationships between these trait groups occurred in wild‐caught butterflies captured during the warmest months and in lab‐reared butterflies reared under summer‐like conditions, whereas these traits were often more weakly related in cool‐season or cool, short‐day reared butterflies. In the wild‐caught butterflies, this may simply reflect the fact that temperature variation (and thus allocation variation) is less in warmer periods. However, in the lab experiment, all low‐temperature butterflies were reared under the same conditions. The resource acquisition (and assimilation) strategies of these butterflies may themselves be plastic in response to temperature and/or resource abundance (Ngoma et al., 2017). Although tyrosine is not an abundant amino acid, relatively speaking, in *Pieris* hostplants it and other amino acids do vary among species and cultivars, seasonally and even among tissue types (Gomes & Rosa, 2001; Kumar et al., 2017; Oliveira et al., 2008; Shawon et al., 2018) and fitness proxies such as larval growth rates and adult size, and even feeding behaviors and resource assimilation strategies, can be affected by host plant attributes (Chen et al., 2004; Hwang et al., 2008; Slansky & Feeny, 1977). Furthermore, hostplant choice behaviors in female cabbage white butterflies can be quite sophisticated (Jaumann & Snell‐Rood, 2017; Steck et al., 2023). Thus despite being crucifer specialists (i.e. family Brassicaceae), they might have considerable scope for adaptively moderating the trade‐offs proposed here under differing resource scenarios.

## AUTHOR CONTRIBUTIONS

**Andrew M. Stoehr:** Conceptualization (lead); data curation (lead); formal analysis (lead); funding acquisition (lead); investigation (lead); methodology (lead); project administration (lead); resources (lead); software (lead); supervision (lead); validation (lead); visualization (lead); writing – original draft (lead); writing – review and editing (lead). **Katelyn Glaenzer:** Conceptualization (supporting); investigation (supporting); methodology (supporting). **Devin VanWanzeele:** Conceptualization (supporting); investigation (supporting); methodology (supporting). **Samantha Rumschlag:** Formal analysis (supporting); writing – original draft (supporting); writing – review and editing (supporting).

## CONFLICT OF INTEREST STATEMENT

The authors declare no conflicts of interest.

## STATEMENT ON INCLUSION

Our study was conducted by researchers from the general region (the midwestern United States) where the research was conducted, though none of the researchers are indigenous to the area. Efforts were made to include among the authors and non‐authoring assistants a diverse group and collection of perspectives.

## Supporting information


Appendix S1.

## Data Availability

Data files and code used to analyze the data and produce the figures for the manuscript are available at the following links: https://figshare.com/projects/Resource‐based_trade‐offs_in_cabbage_white_butterflies/184162. The files may be cited as follows: Stoehr (2024a, 2024b, 2024c, 2024d, 2024e, 2024f).
