# Supplementary material for: Resource‐based trade‐offs and the adaptive significance of seasonal plasticity in butterfly wing melanism
Source: Ecol Evol. 2024 May 1;14(5):e11309. doi: 10.1002/ece3.11309 (PMC11063730; doi:10.1002/ece3.11309)
Supplement: Supplementary file 1 — Appendix S1. [file ECE3-14-e11309-s001.pdf]

## Supporting Information: Table S1 & Appendix 1

**Table S1.** Ingredients lists for maintenance, low tyrosine (LT) and high tyrosine (HT) diets

| Ingredient[1] (units) | Maintenance | Low Tyrosine (LT) | High Tyrosine (HT) |
|---|---|---|---|
| Water (ml) | 800 | 800 | 800 |
| Wheat germ (g) | 60 | 60 | 60 |
| Casein (g) | 27 | 13.5 | 13.5 |
| Sucrose (g) | 24 | 24 | 24 |
| Cabbage Powder (g) | 15 | 15 | 15 |
| Agar (g) | 15 | 15 | 15 |
| Torula yeast (g) | 12 | 12 | 12 |
| Vitamins (Vanderzant) (g) | 10.5 | 10.5 | 10.5 |
| Salts (Beck's) (g) | 9 | 9 | 9 |
| Flax oil[2] (ml) | 4.55 | 4.55 | 4.55 |
| Cholesterol (g) | 3.6 | 3.6 | 3.6 |
| Ascorbic acid (g) | 3 | 3 | 3 |
| Sorbic acid (g) | 1.5 | 1.5 | 1.5 |
| Methyl paraben (g) | 0.75 | 0.75 | 0.75 |
| Streptomycin (g) | 0.175 | 0.175 | 0.175 |
| Cellulose (g) | 0 | 13.5 | 13.5 |
| L-tyrosine[3] (g) | 0 | 0 | 3 |

[1] Source: Frontier Agriculture Sciences (Newark, DE, USA), with the exception of [2]flax oil (Meijer Supermarket, Carmel, IN, USA) and [3]L-tyrosine (Sigma-Aldrich, St. Lous, MO, USA)

**Appendix S1: Estimates of protein and tyrosine content of cabbage (*Brassica oleracea*) and artificial diets:**

To estimate the protein and tyrosine content of the artificial diets, we considered the contributions provided by the cabbage powder, casein, wheat germ, and yeast. Estimates of protein and tyrosine content for wheat germ and casein come from Moore (1985). We used the data from Øverland et al. (2013) for the yeast *Candida utilis* to estimate protein and tyrosine contributions of the Torula yeast. Estimates for the cabbage powder come from the USDA National Nutrient Database.

**Cabbage powder:** According to the USDA National Nutrient Database for Standard Reference, Release 28, 100g of cabbage contains 92.18g of water and thus 7.82g of dry mass. The dry mass contains 1.28g of protein, which is 16.37%. The dry mass contains 0.019g of tyrosine, which is 0.24% tyrosine. These estimates are similar to those for dried cabbage powder (14.0% protein, 0.17% tyrosine) from Mustafa and Baurhoo (2017). The 15g of cabbage powder in our diet therefore provides approximately 2.46g of protein and 0.036g of tyrosine.

**Casein:** According to Moore (1985), casein is 97.04% protein and 4.91% tyrosine. Therefore, the 13.5g of casein in our diet provides 13.1g of total protein and 0.663g of tyrosine.

**Wheat germ:** Moore (1985) reports that wheat germ is 24.9% protein and 0.72% tyrosine; the 60g of wheat germ in our diet therefore provides 14.94g or protein and 0.432g of tyrosine.

**Yeast:** According to Øverland *et al.* (2013), dried yeast *Candida utilisis* is 56% protein and the protein is 3.59% tyrosine. Our Torula yeast powder therefore provides about 6.72g of protein and 0.241g of tyrosine.

The sum of these quantities, which should represent virtually all of the protein and tyrosine in the low tyrosine (LT) diet, comes to 37.215g of protein and 1.372g of tyrosine. The dry mass (i.e. all ingredients except water) of a batch of diet (low tyrosine diet) is 186.075g. The high tyrosine diet is identical, except that 3.0g of tyrosine was added, which in turn increases the total dry mass to 189.075g. Our estimates of dietary protein and tyrosine percentages are based on these values. That is, (37.215g/186.075g)*100 = 20% protein (LT) and (1.372g/186.075)*100 = 0.737% tyrosine (LT). Estimates for the HT diet come from ((37.215 + 3.0)/189.075)*100 = 21.3% protein and ((1.372 + 3.0)/189.075)*100 = 2.31% tyrosine.

**References for Appendix S1**

Moore, R.F. (1985) Artificial diets: development and improvement. *Handbook of Insect Rearing Volume I* (ed. by P. Singh and R.F. Moore), pp. 67-83. Elsevier, Amsterdam.

Mustafa, A.F. & Baurhoo, B. (2017) Evaluation of dried vegetables residues for poultry: II. Effects of feeding cabbage leaf residues on broiler performance, ileal digestibility and total tract nutrient digestibility. *Poultry Science*, **96**, 681-686.

Øverland, M., Karlsson, A., Mydland, L.T., Romarheim, O.H. & Skrede, A. (2013) Evaluation of *Candida utilis*, *Kluyveromyces marxianus* and *Saccharomyces cerevisiae* yeasts as protein sources in diets for Atlantic salmon (*Salmo salar*). *Aquaculture*, **402-403**, 1-7.

USDA National Nutrient Database: https://fdc.nal.usda.gov/fdc-app.html#/food-details/169975/nutrients

**Fig. S1 –** The range of recorded daily mean temperatures increases as the time period under consideration increases. Shown are the mean and standard error of the mean for several time periods, of varying lengths, and across the years of the study. The mean temperature range for the longest time period (246 days) was calculated from the values for each year. For shorter time periods, there were multiple periods per year; the means and standard errors depict the values calculated using all of those data.

Fig. S1

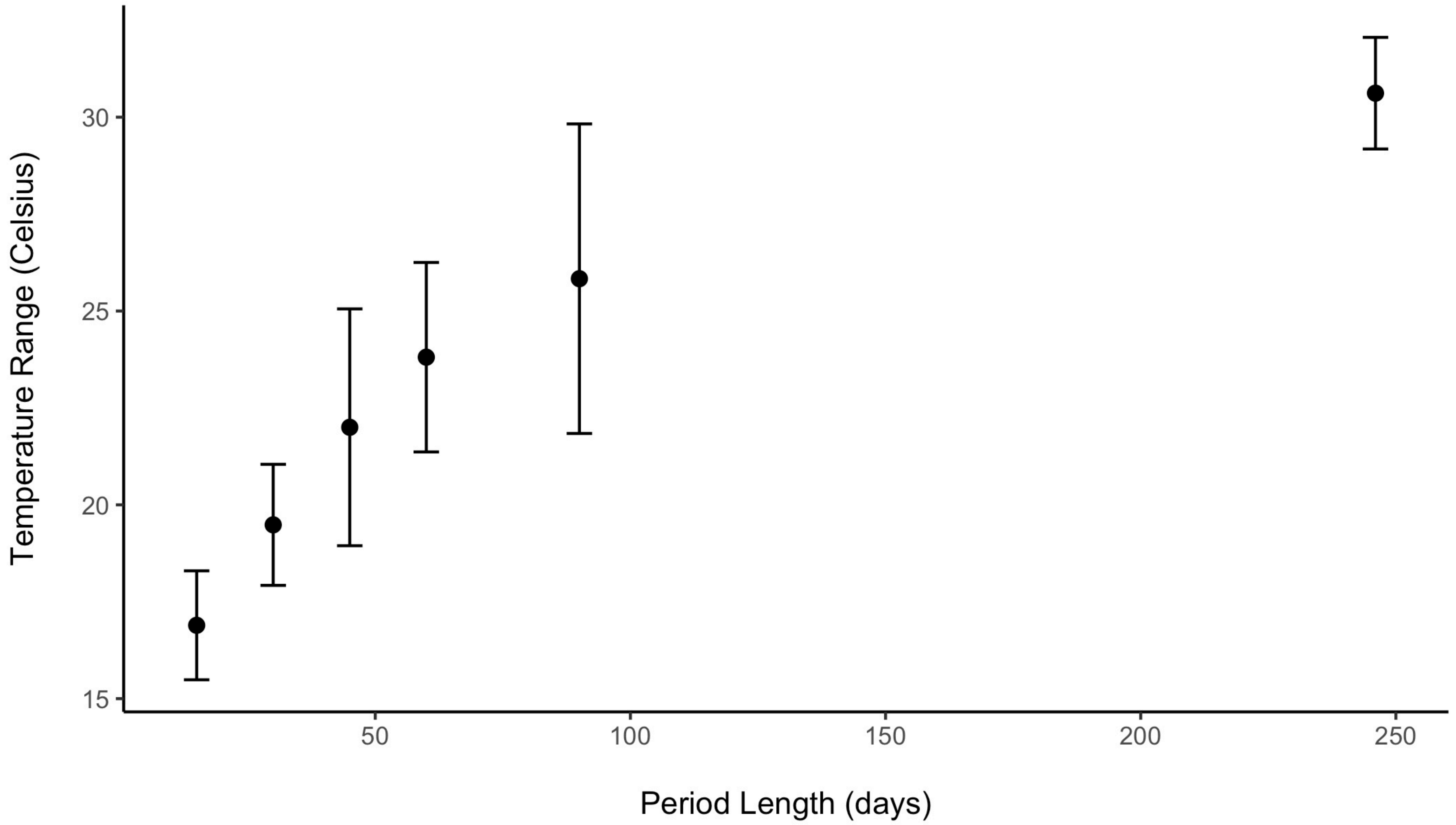
Temperature Range (Celsius)
15
20
25
30
50
100
150
200
250
Period Length (days)

**Supporting Information: Supplementary Tables S2a,b,c – S11a,b,c**

The following tables contain the F-test (i.e. ANOVA) results (table a), regression coefficients (table b), simple slopes (table c) and, in two cases, estimated marginal means (table d) for each model. All linear models used Type 3 (i.e. adjusted) sums-of-squares and, when categorical variables were present, sum contrasts (i.e. deviation or effect coding) using the **car** package for R. The simple slopes show the change in spot size with change in shading melanism at the mean value (0, because it is standardized) of wing size (variable "size"). Simple slopes and estimated marginal means were obtained with the *emtrends* and *emmeans* functions, respectively, of the **emmeans** R package. Regression coefficients, simple slopes, standard errors (Std. Error), 95% confidence intervals (95% CI) F-, t- and P-values are reported to three decimal places except where P-values were below 0.001 and 0.0001. (Note that all analyses shown for the wild-caught butterflies use the approximately 30-day periods as described in the main text. Analyses for all of the other time periods (15, 45, 60, and 90 days) are not shown, but are reproducible from the data and code provided.) The reference levels in the regression coefficient tables for the categorical variables of temperature/photoperiod and dietary tyrosine level are cool, short-day and high tyrosine (HT), respectively.

Tables S2a-c: Wild-caught males, across all seasonal periods

Table S2a: F-test results for wild-caught males, across all periods

| *Model: spots ~ size + shading* | | | |
|---|---|---|---|
| **Variable** | **F-value** | **df** | **P-value** |
| intercept | 0.344 | 1, 2046 | 0.558 |
| size | 587.628 | 1, 2046 | < 0.0001 |
| shading | 724.352 | 1, 2046 | < 0.0001 |

Table S2b: Regression coefficients (Estimates) for wild-caught males, across all periods

| *Model: spots ~ size + shading* | | | | |
|---|---|---|---|---|
| **Variable** | **Estimate** | **Std. Error** | **t-value** | **P-value** |
| intercept | -0.001 | 0.016 | -0.586 | 0.558 |
| size | 0.405 | 0.017 | 24.241 | < 0.0001 |
| shading | -0.452 | 0.017 | -26.914 | < 0.0001 |

Table S2c: Simple slope for wild-caught males, across all periods

| *Model: spots ~ size + shading* | | | |
|---|---|---|---|
| **Variable** | **Slope** | **Std. Error** | **95% CI** |
| shading | -0.452 | 0.017 | -0.485, -0.419 |

Tables S3a-c: Wild-caught males, within seasonal periods

Table S3a: F-test results for wild-caught males, within periods

| *Model: spots ~ size + period + shading + period:shading* | | | |
|---|---|---|---|
| **Variable** | **F-value** | **df** | **P-value** |
| intercept | 31.254 | 1, 2032 | < 0.0001 |
| size | 358.309 | 1, 2032 | < 0.0001 |
| period | 45.780 | 7, 2032 | < 0.0001 |
| shading | 2.761 | 1, 2032 | 0.097 |
| period:shading | 5.166 | 7, 2046 | < 0.0001 |

Table S3b: Regression coefficients for wild-caught males, within periods

| *Model: spots ~ size + period + shading + period:shading* | | | | |
|---|---|---|---|---|
| **Variable** | **Estimate** | **Std. Error** | **t-value** | **P-value** |
| intercept | -0.214 | 0.038 | -5.59 | < 0.0001 |
| size | 0.324 | 0.017 | 18.929 | < 0.0001 |
| Apr-May | -1.190 | 0.117 | -10.197 | < 0.0001 |
| Aug-Sep | 0.666 | 0.057 | 11.704 | < 0.0001 |
| Jul-Aug | 0.939 | 0.121 | 7.733 | < 0.0001 |
| Jun-Jul | 0.398 | 0.0878 | 4.531 | < 0.0001 |
| Mar-Apr | -1.055 | 0.163 | -6.456 | < 0.0001 |
| May-Jun | -0.071 | 0.070 | -1.013 | 0.311 |
| Oct-Nov | -0.056 | 0.101 | -0.554 | 0.580 |
| shading | 0.065 | 0.039 | 1.662 | 0.097 |
| Apr-May:shading | -0.079 | 0.064 | -1.235 | 0.217 |
| Aug-Sep:shading | 0.280 | 0.088 | 3.167 | 0.002 |
| Jul-Aug:shading | 0.423 | 0.160 | 2.654 | 0.008 |
| Jun-Jul:shading | -0.127 | 0.118 | -1.073 | 0.283 |
| Mar-Apr:shading | -0.123 | 0.076 | -1.621 | 0.105 |
| May-Jun:shading | -0.434 | 0.099 | -4.394 | < 0.0001 |
| Oct-Nov:shading | 0.065 | 0.116 | 0.560 | 0.576 |

Table S3c: Simple slopes for wild-caught males, within periods

| *Model: spots ~ size + period + shading + period:shading* | | | |
|---|---|---|---|
| **Variable** | **Slope** | **Std. Error** | **95% CI** |
| Mar-Apr | -0.057 | 0.075 | -0.205, 0.090 |
| Apr-May | -0.014 | 0.059 | -0.130, 0.102 |
| May-Jun | -0.368 | 0.105 | -0.574, -0.163 |
| Jun-Jul | -0.061 | 0.129 | -0.315, 0.192 |
| Jul-Aug | 0.489 | 0.179 | 0.137, 0.839 |
| Aug-Sep | 0.345 | 0.092 | 0.165, 0.525 |
| Sep-Oct | 0.059 | 0.060 | -0.059, 0.177 |
| Oct-Nov | 0.132 | 0.127 | -0.118, 0.379 |

Tables S4a-c: Wild-caught females, across all seasonal periods

Table S4a: F-test results for wild-caught females, across all periods

| *Model: spots ~ size + shading* | | | |
|---|---|---|---|
| **Variable** | **F-value** | **df** | **P-value** |
| intercept | 0.002 | 1, 1171 | 0.962 |
| size | 384.809 | 1, 1171 | < 0.0001 |
| shading | 62.585 | 1, 1171 | < 0.0001 |

Table S4b: Regression coefficients for wild-caught females, across all periods

| *Model: spots ~ size + shading* | | | | |
|---|---|---|---|---|
| **Variable** | **Estimate** | **Std. Error** | **t-value** | **P-value** |
| intercept | -0.001 | 0.025 | -0.047 | 0.962 |
| size | 0.493 | 0.025 | 19.617 | < 0.0001 |
| shading | -0.198 | 0.025 | -7.911 | < 0.0001 |

Table S4c: Simple slope for wild-caught females, across all periods

| *Model: spots ~ size + shading* | | | |
|---|---|---|---|
| **Variable** | **Slope** | **Std. Error** | **95% CI** |
| shading | -0.198 | 0.025 | -0.248, -0.149 |

Tables S5a-c: Wild-caught females, within seasonal periods

Table S5a: F-test results for wild-caught females, within periods

| *Model: spots ~ size + period + shading + period:shading* | | | |
|---|---|---|---|
| **Variable** | **F-value** | **df** | **P-value** |
| intercept | 64.054 | 1, 1157 | < 0.0001 |
| size | 210.264 | 1, 1157 | < 0.0001 |
| period | 37.662 | 7, 1157 | < 0.0001 |
| shading | 46.816 | 1, 1157 | < 0.0001 |
| period:shading | 1.646 | 7, 1157 | 0.119 |

Table S5b: Regression coefficients for wild females, within periods

| *Model: spots ~ size + period + shading + period:shading* | | | | |
|---|---|---|---|---|
| **Variable** | **Estimate** | **Std. Error** | **t-value** | **P-value** |
| intercept | -0.381 | 0.048 | -8.003 | < 0.0001 |
| size | 0.350 | 0.024 | 14.500 | < 0.0001 |
| Apr-May | -1.187 | 0.196 | -6.065 | < 0.0001 |
| Aug-Sep | 0.775 | 0.078 | 9.905 | < 0.0001 |
| Jul-Aug | 1.115 | 0.125 | 8.890 | < 0.0001 |
| Jun-Jul | 0.736 | 0.092 | 7.972 | < 0.0001 |
| Mar-Apr | -1.791 | 0.193 | -9.295 | < 0.0001 |
| May-Jun | -0.095 | 0.092 | -1.034 | 0.302 |
| Oct-Nov | 0.200 | 0.105 | 1.910 | 0.056 |
| shading | 0.278 | 0.041 | 6.769 | < 0.0001 |
| Apr-May:shading | -0.215 | 0.126 | -1.705 | 0.089 |
| Aug-Sep:shading | 0.07368 | 0.102 | 0.721 | 0.471 |
| Jul-Aug:shading | 0.20027 | 0.144 | 1.395 | 0.163 |
| Jun-Jul:shading | -0.15664 | 0.108 | -1.447 | 0.148 |
| Mar-Apr:shading | 0.03903 | 0.094 | 0.416 | 0.678 |
| May-Jun:shading | 0.18253 | 0.105 | 1.736 | 0.083 |
| Oct-Nov:shading | -0.12604 | 0.079 | -1.592 | 0.112 |

Table S5c: Simple slopes for wild-caught females, within periods

| *Model: spots ~ size + period + shading + period:shading* | | | |
|---|---|---|---|
| **Variable** | **Slope** | **Std. Error** | **95% CI** |
| Mar-Apr | 0.317 | 0.098 | 0.125, 0.509 |
| Apr-May | 0.064 | 0.138 | -0.208, 0.335 |
| May-Jun | 0.461 | 0.113 | 0.239, 0.682 |
| Jun-Jul | 0.121 | 0.117 | -0.108, 0.351 |
| Jul-Aug | 0.478 | 0.160 | 0.164, 0.792 |
| Aug-Sep | 0.352 | 0.110 | 0.136, 0.567 |
| Sep-Oct | 0.280 | 0.050 | 0.181, 0.378 |
| Oct-Nov | 0.152 | 0.080 | -0.004, 0.308 |

Tables S6a-c: Males, across all treatment groups in temperature/photoperiod, tyrosine manipulation study

Table S6a: F-test results for males, across all treatments

| *Model: spots ~ size + shading* | | | |
|---|---|---|---|
| **Variable** | **F-value** | **df** | **P-value** |
| intercept | 0.000 | 1,330 | 1.000 |
| size | 61.684 | 1,330 | < 0.0001 |
| shading | 32.463 | 1,330 | < 0.0001 |

Table S6b: Regression coefficients for males, across all treatments

| *Model: spots ~ size + shading* | | | | |
|---|---|---|---|---|
| **Variable** | **Estimate** | **Std. Error** | **t-value** | **P-value** |
| intercept | 0.000 | 0.049 | 0.000 | 1.000 |
| size | 0.382 | 0.049 | 7.854 | < 0.0001 |
| shading | -0.277 | 0.049 | -5.698 | < 0.0001 |

Table S6c: Simple slope for males, across all treatments

| *Model: spots ~ size + shading* | | | |
|---|---|---|---|
| **Variable** | **Slope** | **Std. Error** | **95% CI** |
| shading | -0.277 | 0.049 | -0.372, -0.181 |

Tables S7a-c: Males, within diets (i.e. tyrosine levels: high tyrosine (HT), low tyrosine (LT)), across temperature/photoperiod rearing treatments

Table S7a: F-test results for males, within diets, across rearing

| *Model: spots ~ size + diet + shading + diet:shading* | | | |
|---|---|---|---|
| **Variable** | **F-value** | **df** | **P-value** |
| intercept | 0.098 | 1, 328 | 0.755 |
| size | 63.724 | 1, 328 | < 0.0001 |
| diet | 3.226 | 1, 328 | 0.073 |
| shading | 24.814 | 1, 328 | < 0.0001 |
| diet:shading | 2.407 | 1, 328 | 0.122 |

Table S7b: Regression coefficients for males, within diets, across rearing

| *Model: spots ~ size + diet + shading + diet:shading* | | | | |
|---|---|---|---|---|
| **Variable** | **Estimate** | **Std. Error** | **t-value** | **P-value** |
| intercept | 0.015 | 0.049 | 0.313 | 0.755 |
| size | 0.398 | 0.050 | 7.983 | < 0.0001 |
| diet (HT) | 0.090 | 0.050 | 1.796 | 0.073 |
| shading | -0.249 | 0.050 | -4.981 | < 0.0001 |
| diet (HT):shading | 0.078 | 0.050 | 1.551 | 0.122 |

Table S7c: Simple slopes for males, within diets, across rearing

| *Model: spots ~ size + diet + shading + diet:shading* | | | |
|---|---|---|---|
| **Variable** | **Slope** | **Std. Error** | **95% CI** |
| HT diet | -0.172 | 0.079 | -0.327, -0.016 |
| LT diet | -0.327 | 0.062 | -0.448, -0.206 |

Tables S8a-c: Males, within temperature/photoperiod rearing treatments (temp/photo) (cool, short-day or warm, long-day), across diets (i.e. tyrosine levels)

Table S8a: F-test results for males, within rearing, across diets

| *Model: spots ~ size + temp/photo + shading + temp/photo:shading* | | | |
|---|---|---|---|
| **Variable** | **F-value** | **df** | **P-value** |
| intercept | 7.878 | 1, 328 | 0.005 |
| size | 62.947 | 1, 328 | < 0.0001 |
| temp/photo | 25.693 | 1, 328 | < 0.0001 |
| shading | 14.104 | 1, 328 | < 0.001 |
| temp/photo:shading | 13.150 | 1, 328 | < 0.001 |

Table S8b: Regression coefficients for males, within rearing, across diets

| *Model: spots ~ size + temp/photo + shading + temp/photo:shading* | | | | |
|---|---|---|---|---|
| **Variable** | **Estimate** | **Std. Error** | **t-value** | **P-value** |
| intercept | 0.614 | 0.219 | 2.807 | 0.005 |
| size | 0.372 | 0.047 | 7.934 | < 0.0001 |
| temp/photo (cool, short-day) | -1.109 | 0.219 | -5.069 | < 0.0001 |
| shading | 1.141 | 0.304 | 3.756 | < 0.001 |
| temp/photo (cool, short-day):shading | -1.102 | 0.304 | -3.626 | < 0.001 |

Table S8c: Simple slopes for males, within rearing, across diets

| *Model: spots ~ size + temp/photo + shading + temp/photo:shading* | | | |
|---|---|---|---|
| **Variable** | **Slope** | **Std. Error** | **95% CI** |
| cool, short-day | 0.040 | 0.124 | -0.205, 0.284 |
| warm, long-day | 2.243 | 0.595 | 1.073, 3.413 |

Tables S9a-c: Females, across all treatment groups in temperature/photoperiod, tyrosine manipulation study

Table S9a: F-test results for females, across all treatments

| *Model: spots ~ size + shading* | | | |
|---|---|---|---|
| **Variable** | **F-value** | **df** | **P-value** |
| intercept | 0.000 | 1,298 | 1.000 |
| size | 84.328 | 1,298 | < 0.0001 |
| shading | 22.307 | 1,298 | < 0.0001 |

Table S9b: Regression coefficients for females, across all treatments

| *Model: spots ~ size + shading* | | | | |
|---|---|---|---|---|
| **Variable** | **Estimate** | **Std.Error** | **t-value** | **P-value** |
| intercept | 0.000 | 0.049 | 0.000 | 1.000 |
| size | 0.453 | 0.049 | 9.183 | < 0.0001 |
| shading | -0.233 | 0.049 | -4.723 | < 0.0001 |

Table S9c: Simple slope for females, across all treatments

| *Model: spots ~ size + shading* | | | |
|---|---|---|---|
| **Variable** | **Slope** | **Std. Error** | **95% CI** |
| shading | -0.233 | 0.049 | -0.330, -0.136 |

Tables S10a-c: Females, within diets (i.e. tyrosine levels: high tyrosine (HT), low tyrosine (LT)), across temperature/photoperiod rearing treatments

Table S11a: F-test results for females, within diets, across rearing

*Model: spots ~ size + diet + shading + diet:shading*

| **Variable** | **F-value** | **df** | **P-value** |
|---|---|---|---|
| intercept | 0.576 | 1, 296 | 0.448 |
| size | 117.512 | 1, 296 | < 0.0001 |
| diet | 25.691 | 1, 296 | < 0.0001 |
| shading | 19.112 | 1, 296 | < 0.0001 |
| diet:shading | 1.124 | 1, 296 | 0.290 |

Table S10b: Regression coefficients for females, within diets, across rearing

*Model: spots ~ size + diet + shading + diet:shading*

| **Variable** | **Estimate** | **Std.Error** | **t-value** | **P-value** |
|---|---|---|---|---|
| intercept | 0.036 | 0.048 | 0.759 | 0.448 |
| size | 0.560 | 0.052 | 10.840 | < 0.0001 |
| diet (HT) | 0.262 | 0.052 | 5.069 | < 0.0001 |
| shading | -0.222 | 0.051 | -4.372 | < 0.0001 |
| diet (HT):shading | 0.053 | 0.050 | 1.060 | 0.290 |

Table S10c: Simple slopes for females, within diets, across rearing

*Model: spots ~ size + diet + shading + diet:shading*

| **Variable** | **Slope** | **Std. Error** | **95% CI** |
|---|---|---|---|
| HT diet | -0.168 | 0.083 | -0.332, -0.005 |
| LT diet | -0.275 | 0.057 | -0.388, -0.162 |

Tables S11a-c: Females, within temperature/photoperiod rearing treatments (temp/photo) (cool, short-day or warm, long-day), across diets (i.e. tyrosine levels)

Table S11a: F-test results for females, within rearing, across diet

*Model: spots ~ size + temp/photo + shading + temp/photo:shading*

| Variable | F-value | df | P-value |
|---|---|---|---|
| intercept | 26.861 | 1, 296 | < 0.0001 |
| size | 61.506 | 1, 296 | < 0.0001 |
| temp/photo | 55.576 | 1, 296 | < 0.0001 |
| shading | 33.263 | 1, 296 | < 0.0001 |
| temp/photo:shading | 23.625 | 1, 296 | < 0.0001 |

Table S11b: Regression coefficients for females, across diets/rearing, within temps

*Model: spots ~ size + temp/photo + shading + temp/photo:shading*

| Variable | Estimate | Std. Error | t-value | P-value |
|---|---|---|---|---|
| intercept | 0.959 | 0.185 | 5.183 | < 0.0001 |
| size | 0.367 | 0.047 | 7.843 | < 0.0001 |
| temp/photo (cool, short-day) | -1.396 | 0.187 | -7.455 | < 0.0001 |
| shading | 1.044 | 0.181 | 5.767 | < 0.0001 |
| temp/photo (cool, short-day):shading | -0.875 | 0.180 | -4.861 | < 0.0001 |

Table S11c: Simple slopes for females, across diets/rearing, within temps

*Model: spots ~ size + temp/photo + shading + temp/photo:shading*

| Variable | Slope | Std. Error | 95% CI |
|---|---|---|---|
| cool, short-day | 0.169 | 0.097 | -0.023, 0.361 |
| warm, long-day | 1.920 | 0.348 | 1.235, 2.604 |
